# Evaluation of ankle fracture classification systems in 193 trimalleolar ankle fractures

**DOI:** 10.1007/s00068-022-01959-2

**Published:** 2022-03-29

**Authors:** Patrick Pflüger, Felix Harder, Karoline Müller, Peter Biberthaler, Moritz Crönlein

**Affiliations:** 1 Department of Trauma Surgery, Klinikum Rechts der Isar, Technical University of Munich, Ismaninger Str. 22, 81675 Munich, Germany; 2 Institute of Diagnostic and Interventional Radiology, Technical University of Munich, School of Medicine, Munich, Germany

**Keywords:** Ankle fracture, Medial malleolus, Posterior malleolus, Classifications, Reliability

## Abstract

**Purpose:**

Different classification systems have been developed for ankle fractures. In recent years, the posterior malleolus has gained in importance and led to computed tomography (CT)-based classification systems. The aim of the study was to analyse their reliability, fracture patterns and influence on treatment strategy.

**Methods:**

Patients with a trimalleolar ankle fracture treated between 2011 and 2020 with preoperative radiographs and CT images were included. The blinded images were independently classified by three reviewers according to the AO/OTA, Herscovici, Bartoníček, Mason and Haraguchi classifications. The interobserver reliability was calculated by Fleiss' kappa (κ). CT images were analysed to determine the dimensions of the posterior malleolus fragments. Patient registries were reviewed regarding the treatment data.

**Results:**

A total of 193 patients were included. The AO/OTA classification showed almost perfect inter- and intraobserver reliability (Fleiss’ κ = 0.86, 95% CI 0.82–0.90). Regarding the posterior malleolus, the Bartoníček classification demonstrated the highest reliability (Fleiss’ κ = 0.78, 95% CI 0.73–0.83). The Herscovici classification only reached moderate reliability for medial malleolus fractures (Fleiss' κ = 0.59, 95% CI 0.54–0.65). There was a trend towards direct fixation of the posterior malleolus in the last 3 years of the observation period (OR: 2.49, 95% CI 1.03–5.99).

**Conclusion:**

In trimalleolar ankle fractures, the AO/OTA classification is a reliable system to characterize the type of fracture, but it fails to provide solid information about the posterior malleolus. Nowadays, treatment recommendations for trimalleolar ankle fractures focus on the configuration of the posterior malleolus; therefore, the results of this study advocate the use of the Bartoníček classification as a reliable tool to guide treatment.

## Background

Ankle fractures account for up to 10% of all bone injuries, with an increasing incidence over the last decades [1, 2]. Trimalleolar ankle fractures form one of the most complex entities by involving the distal fibula (lateral malleolus), medial malleolus and posterior malleolus. Different classification systems have been developed over time, depending on the mechanism of injury, biomechanical findings and radiographic evaluations [3]. The pursued objective is to establish a uniform system, enabling a standardized and rational methodology of describing fractures, that provides the ability to code data for clinical interaction and research [4]. The AO/OTA classification represents the most comprehensive classification system and provides an overview, with a focus on the fracture pattern of the fibula [4]. However, important information regarding the configurations of the medial and posterior malleoli are not fully depicted. Continuous research has led to new, more specific classification systems for the medial and posterior malleoli [3]. Herscovici et al. proposed a classification system for fractures of the medial malleolus, which went on to be the most used in recent literature due to its simplicity and usability in clinics [5, 6]. The posterior malleolus has gained importance over the last few years. New evidence regarding the treatment is rising [7]. The increased routine use of computed tomography (CT) led to new CT-based classification systems [8–10]. The proposed classifications for posterior malleolus fractures by Haraguchi et al., Mason et al. and Bartoníček et al. are currently used in the literature [3]. This displays the change regarding the decisive factors for successful treatment of ankle fractures, overcoming the previously existing assumption that only the extent and displacement of the posterior malleolus fragment is relevant [11].

In general, a trimalleolar ankle fracture is considered unstable and treatment is performed operatively [3]. There is no debate whether to stabilize the fibula in these ankle fractures, but the optimal treatment of fractures of the medial and posterior malleolus remains unclear [3, 6]. To date, there exists no uniform treatment recommendation based on a classification system. Therefore, an attempt has been made to develop an algorithm for the treatment of fractures of the posterior malleolus based on the Bartoníček classification [12].

The reliability of ankle classification systems in trimalleolar ankle fractures has not been investigated before. Only a few studies exist either investigating the reliability of the Herscovici or Haraguchi classification systems in the current literature [13, 14].

Therefore, the aim of this study was to analyse the reliability of the pre-existing classification systems, fracture patterns and influence on the treatment strategy.

## Methods

A retrospective analysis of patients treated for a trimalleolar ankle fracture from 2011 to 2020 was performed at a level I orthopaedic trauma department. Only patients with preoperative radiographs and CT images were included. Patient records were reviewed regarding the operative procedure. For the treatment analysis, only patients with complete operative records were included.

The study was approved by the local regulatory committee (No: 600/21 S, Technical University of Munich, Germany).

### Radiographic analysis and classification

Blinded radiographs were independently classified by three investigators of our interdisciplinary research group with profound knowledge in musculoskeletal imaging (two foot and ankle surgeons and one radiologist). The observers were provided with illustrations of the respective classifications during the review. For the intraobserver reliability, the blinded radiographs were classified again after 3 months by the three investigators.

**Fig. 1:**
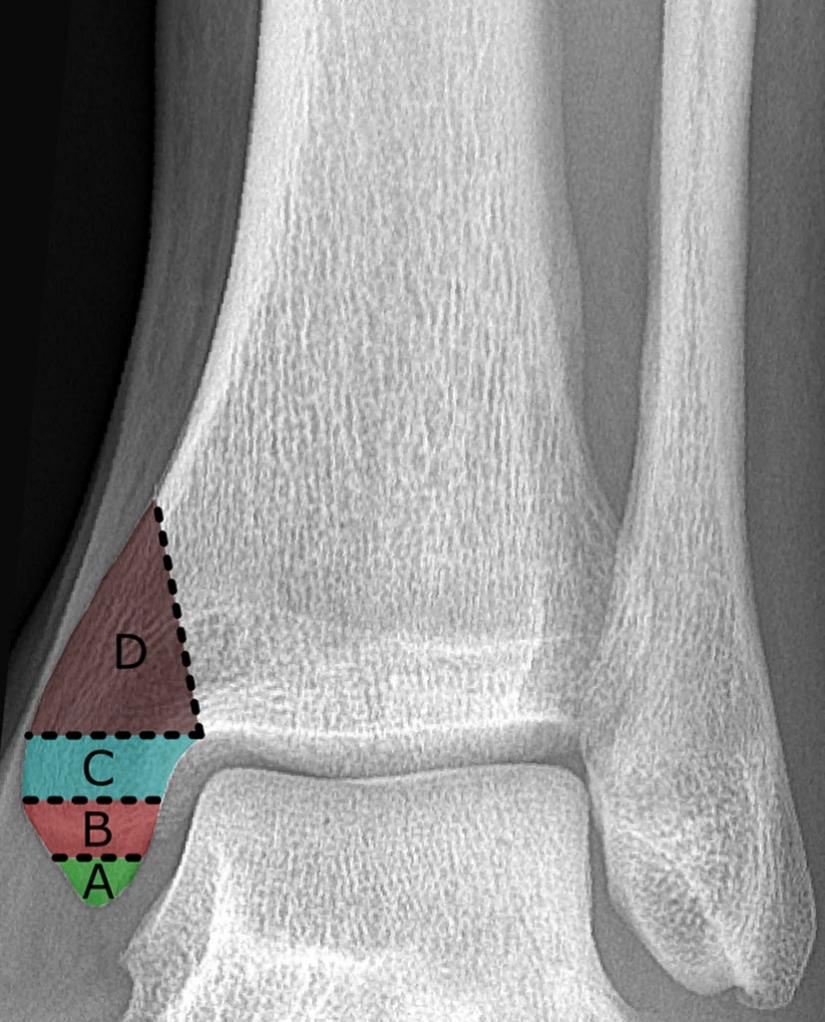
AP ankle radiograph illustrating the Herscovici classification. **A** Avulsions at the tip of the medial malleolus. **B** Fractures between the tip and the plafond. **C** Fractures at the level of the plafond. **D** Oblique-vertical fractures from the plafond [5]

Fractures of the medial malleolus were classified according to Herscovici et al. on anterior–posterior (AP) ankle radiographs [5]. According to this classification system, fractures of the medial malleolus can be divided into four types (A-D) of fractures (Fig. 1). Furthermore, fractures of the medial malleolus were differentiated into the following: anterior collicular fracture, posterior collicular fracture, and anterior and posterior collicular fractures.

**Fig. 2:**
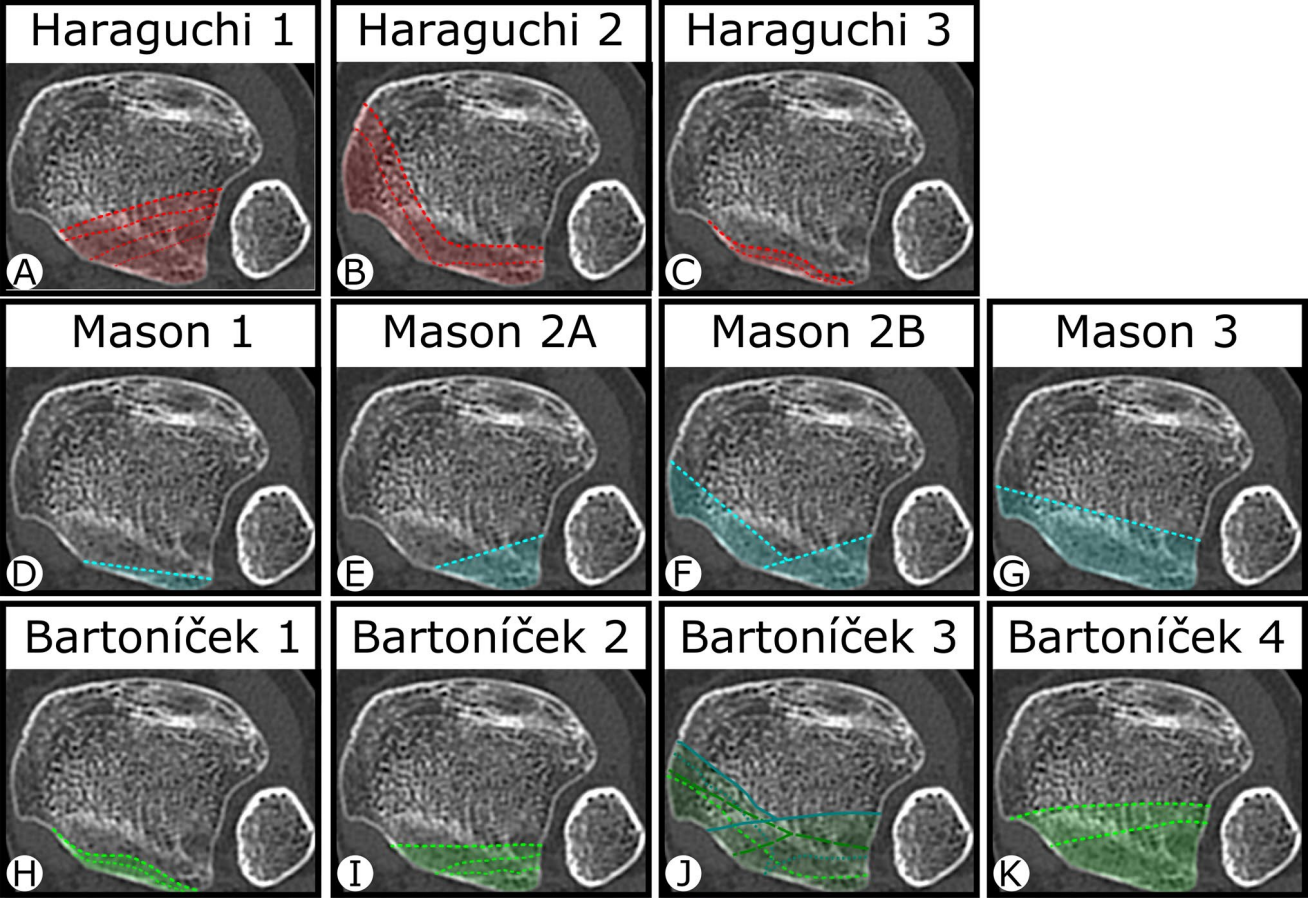
Axial CT sections of the posterior malleolus illustrating the Haraguchi, Mason and Bartoníček classifications. **A** Haraguchi 1: Posterolateral-oblique fracture involving the posterolateral corner of the tibial plafond. **B** Haraguchi 2: Transverse medial-extension fracture from the fibula notch of the tibia. **C** Haraguchi 3: Small shell-shaped fragments at the posterior lip of the tibial plafond [10]. **D** Mason 1: Extra-articular posterior malleolar fracture. **E** Mason 2A: Fracture of the posterolateral triangle of the tibia extending into the incisura. **F** Mason 2B: Posterolateral fracture with secondary fragment on the posteromedial aspect. **G**: Mason 3: Fracture line involving the whole posterior plafond [9]. **H** Bartoníček 1: Extraincisural fracture with an intact fibular notch. **I** Bartoníček 2: Posterolateral fragment extending into the fibular notch. **J** Bartoníček 3: Posteromedial, two-part fragment involving the medial malleolus. **K** Bartoníček 4: Large, posterolateral triangular fragment [8]

The posterior malleolus was classified with CT sections according to Haraguchi et al., Mason et al. and Bartoníček et al. (Fig. 2) [8–10]. Haraguchi et al. differentiated posterior malleolar fragments with transverse CT sections into three different entities (type 1–3) [10]. Mason et al. modified the Haraguchi classification, indicating the severity and pathomechanism of the fracture (type 1, 2A, 2B, or 3) [9]. Bartoníček et al. proposed the most differentiated classification system for the posterior malleolus, taking into consideration the stability of the tibiotalar joint and the integrity of the fibular notch (type 1–5) [8].

The size of the posterior malleolus fragment and the tibial diameter were measured in transverse CT sections, as well as the height of the fragment in the corresponding sagittal CT section. Furthermore, the proportion (%) of the posterior malleolus fragment in relation to the tibial diameter, as well as the area of the posterior malleolus fragment (on the simplified basis of a rectangular triangle) was calculated.

The AO/OTA classification system was used to define the trimalleolar ankle fracture with all involved malleoli more accurately [4]. Decisive for the AO/OTA classification is the type of fibula fracture in relation to the syndesmosis (type A/B/C). Fibula fractures with a fracture of the posterolateral rim and medial malleolus can be classified as AO/OTA type 44B2.3, 44B3.3, 44C1.3 and C2.3. Fractures of the lateral malleolus with a posteromedial fracture can be classified as AO/OTA type 44A3.2 and 44A3.3.

### Treatment

The surgical records were reviewed regarding the type of operative treatment, date and implants. The following data was analysed: need for external fixation, implant type (fibula, medial malleolus or posterior malleolus), lag screw fibula, direct or indirect fixation of the posterior malleolus (indirect screw or direct screw/plate osteosynthesis) and necessity of a trans-syndesmotic fixation. For medial malleolus fractures, the surgical procedures were differentiated into screw osteosynthesis, tension band wiring/k-wires and plate osteosynthesis. In a second step, an analysis was performed as to whether the classification of the involved malleolus or the size of the fragment influenced the type of implant and operative procedure.

### Statistics

Data are presented as medians (interquartile ranges). RStudio (RStudio Team (2020). RStudio: Integrated Development Environment for R. RStudio, PBC, Boston, MA URL http://www.rstudio.com/) was used for data processing, and a p value < 0.05 was considered statistically significant.

Due to the high number of patients, a quantile–quantile (Q-Q) plot was used to assess normality. As appropriate, the nonparametric Mann–Whitney U test or the parametric t-test was used to assess significant differences between two groups, and the Kruskal–Wallis test or analysis of variance (ANOVA) was used in cases of more than two groups. Post hoc analysis was calculated with Bonferroni correction. The reliability of the classification system was determined using Fleiss' kappa (κ) coefficient of agreement (with 95% confidence intervals) with the results of observers (interobserver reliability). For the intraobserver reliability kappa (κ) coefficient of agreement (with 95% confidence intervals) was calculated. κ values were interpreted according to Landis and Koch [15].

To calculate the strength and association of two categorical variables, the OR and 95% CI was calculated. The Chi-squared test was used to assess significant differences between categorial variables.

## Results

A total of 211 patients with a trimalleolar ankle fracture were treated from 2011 to 2020 and 193 patients included for radiographic analysis. 179 patients had complete operative records and were available for further analysis regarding their surgical treatment.

### AO/OTA classification

An overview of the included trimalleolar ankle fractures is illustrated in Table 1. The majority of the patients showed trans-syndesmotic fibula fractures, with a medial injury, and fracture of the posterolateral rim (AO/OTA type 44B3.3 and 44B3.2). An avulsion of the anterior tubercle of the fibula (Le Fort-Wagstaffe tubercle) was evident in 66% of the trimalleolar ankle fractures, and a fracture of the anterior tubercle of the tibia (Chaput’s tubercle) was evident in 14% of the trimalleolar ankle fractures.

**Table 1:** Overview of included trimalleolar ankle fractures

AO/OTA classification		Number (*n*)
A	A3.2	1
	A3.3	1
B	B3.2	44
	B3.2n	5
	B3.2o	19
	B3.2on	1
	B3.3	23
	B3.3n	1
	B3.3o	53
	B3.3on	2
C	C1.3	7
	C2.3	33
	C3.3	3

The AO/OTA classification showed an almost perfect inter- and intraobserver reliability, and if one considers only the fibula fracture (Weber type), an even better κ (Tables 2 and 3).

**Table 2:** Interobserver reliability of the different classification systems

Classification	Kappa	95% CI
AO	0.86	[0.82–0.90]
Weber	0.91	[0.83–0.99]
Herscovici	0.59	[0.54–0.65]
Haraguchi	0.70	[0.64–0.75]
Bartoníček	0.78	[0.73–0.83]
Mason	0.61	[0.56–0.66]

**Table 3:** Intraobserver reliability of the different classification systems

Classification	Rater 1	Rater 2	Rater 3
AO	0.81 [0.74;0.88]	0.81 [0.74;0.88]	0.85 [0.78;0.92]
Weber	0.86 [0.78;0.95]	0.93 [0.87;0.99]	0.91 [0.85;0.98]
Herscovici	0.61 [0.52;0.71]	0.64[0.55;0.73]	0.59 [0.50;0.69]
Haraguchi	0.72 [0.63;0.80]	0.74 [0.66;0.83]	0.77 [0.69;0.85]
Bartoníček	0.79 [0.72;0.87]	0.76 [0.68;0.84]	0.81 [0.74;0.88]
Mason	0.63 [0.54;0.72]	0.64 [0.55;0.74]	0.65 [0.56;0.75]

Illustrated are the kappa values with 95% CI of the three different raters

Patients needing an external fixation had a proportionally greater posterior malleolus fragment (*p* < 0.001). AO/OTA type B3.3 and C fractures in comparison to B3.2 fractures were also more likely to receive external fixation (p_B_ = 0.015, p_C_ = 0.027). Lag screws were not significantly more used in simple fibula fractures (B3.2 and C1.3) in comparison to multifragmentary (B3.3 and C2.3) fractures (*p* = 0.66).

### Herscovici classification

Regarding the interobserver reliability, the Herscovici classification for fractures of the medial malleolus reached moderate reliability (Fleiss' κ = 0.59). Intraobserver reliability showed a substantial strength of agreement (Table 3). Classified according to the anatomical landmarks, there were 37 anterior collicular fractures (19%), 9 posterior collicular fractures (5%), 126 anterior and posterior collicular fractures (65%), and 21 fractures that were not classifiable (11%).

For fractures of the medial malleolus, the most common operative procedure was screw osteosynthesis (*n* = 113). Herscovici type D fractures were more likely to have plate osteosynthesis in comparison to type C fractures (*p* = 0.003), but Herscovici classification was not associated with the type of surgical procedure.

### Haraguchi, Mason and Bartoníček classification

For posterior malleolus fractures, the Bartoníček and Haraguchi classification demonstrated substantial reliability, whereas the Mason classification had the lowest Fleiss' κ (Table 2). The intraobersver reliability of the three different classification systems reached also a substantial agreement (Table 3).

The median size of the tibial diameter was 39.6 mm (4.5), the fragment size in transverse CT sections was 9.2 mm (4.2) and the height of the posterior malleolus fragment (sagittal sections) was 20.5 mm (10.1). Table 4 shows the proportion of the posterior malleolus fragment in relation to the tibial diameter. Most commonly, the posterior malleolus fragment covered 21–25% of the tibial diameter. The area of the posterior malleolus fragment was 97.5 mm^2^ (84.4) and most frequently between 50 and 150 mm^2^ (Table 5).

**Table 4:** Proportional size of the posterior malleolus fragment in relation to the tibial diameter

Proportion (posterior malleolus/tibial diameter)	Number (*n*)
0%-10%	11
11%-15%	20
16%-20%	30
21%-25%	54
26%-30%	36
31%-35%	15
> 35%	27

**Table 5:** Area of the posterior malleolus

Area (mm^2^)	Number (*n*)
0–50	45
51–100	54
101–150	60
> 150	34

Regarding the posterior malleolus fragment, the proportional size (in axial sections) was significantly larger in patients with surgical fixation of the posterior malleolus (direct or indirect) in comparison to those without (*p* < 0.001). But, the bare proportional size of the posterior malleolus fragment was not decisive for direct fixation (*n* = 35) (*p* = 1). The same results are present, considering not only the axial size, but also the height of the posterior malleolus fragment (area of the posterior malleolus fragment). Direct fixation of the posterior malleolus fragment did not supersede the necessity of a trans-syndesmotic screw in comparison to indirect fixation (*p* = 1). Considering the Bartoníček classification, direct fixation was not significantly more often performed in fractures involving the fibular notch (*p* = 0.9). Overall, in the last 3 years of the observation period, there was a trend towards direct fixation of the posterior malleolus (OR: 2.49, 95% CI 1.03–5.99).

## Discussion

In this cohort study, 193 patients with trimalleolar ankle fractures were evaluated, showing that the AO/OTA classification is a reliable system to characterize the type of fracture but fails to provide solid information about the medial and posterior malleoli. Nowadays, treatment recommendations for trimalleolar ankle fractures focus on the configuration of the posterior malleolus; therefore, the results of this study advocate the use of the Bartoníček classification as a reliable tool to guide treatment. To our knowledge, the presented study includes the largest number of patients to evaluate the reliability of classification systems in ankle fractures [13, 14].

The AO/OTA classification showed almost perfect reliability (κ = 0.86), and if only considering the fibula fracture (Weber type), an even better interobserver agreement (κ = 0.91). Equivalent strengths of agreement were found for the intraobserver reliability of the Weber- and AO/OTA classification. The bare differentiation of fibula fractures relevant to the tibiofibular syndesmosis (Weber classification) has high inter- and intraobserver reliability [16, 17]. In trimalleolar ankle fractures, the AO/OTA classification provides an overview by coding the type of trimalleolar ankle fracture and describing the involvement of the medial and posterior malleoli. However, the configurations of the medial and posterior malleoli are not represented [4]. The analysis showed that multifragmentary trans-syndesmotic and supra-syndesmotic trimalleolar ankle fractures (AO/OTA type 44B3.3 and 44C) were more likely to receive external fixation. Interpreting this result as an expression of higher instability and therefore higher severity, this finding corresponds to the current literature, reporting a larger number of intraarticular lesions, involvement of the posterior malleolus and worse outcomes in high-grade unstable ankle fractures [14, 18, 19].

The interobserver reliability of the Herscovici classification for fractures of the medial malleolus is moderate, whereas intraobserver reliabilities showed substantial agreements. This is in line with Aitken et al., who also reported moderate interobserver reliability (κ = 0.54) and substantial reproducibility (κ = 0.64) in 130 cases [13]. They concluded that the Herscovici classification needs to be “refined” [13]. The findings of this study support the conclusion that the type of Herscovici fracture did not influence the kind of surgical procedure. This result is not surprising, considering that the Herscovici classification is based on plain AP radiographs and the ideal management of fractures of the medial malleolus is still debatable [5, 6]. In general, unstable displaced fractures are internally fixed, but the type of fixation method depends on fracture configuration, bone quality and surgeons preference [6]. Herscovici et al. differentiated the type of medial malleolus fracture according to the height of the fracture line in AP radiographs, as the extent and configuration of the fracture (e.g., multifragmentary fracture) is not part of the classification system [5]. Liu et al. have taken the fact as an opportunity and developed a CT-based classification for medial malleolus fractures by analysing 121 ankle fractures [20]. According to the findings of their study, fractures of the medial malleolus can be differentiated into six types depending on the involvement of the anterior and posterior colliculi [20]. Following this proposed classification, the distribution of fractures of the medial malleolus according to collicular differentiation is comparable to the investigated patient population. This supports the conclusion of Aitken et al. and Liu et al. to establish a more specific CT-based classification for fractures of the medial malleolus.

The importance of the posterior malleolus has gained enormous scientific attention, since studies have demonstrated a significant influence in functional patient-related outcomes [7, 21]. However, there are only two studies assessing the interobserver reliability of the Haraguchi classification in a representative patient population [9, 14]. In this study, the Haraguchi classification showed substantial interobserver reliability, comparable with the study by Raeder et al. [14]. Mason et al. even reported almost perfect interobserver reliability with the Haraguchi classification in their analysis of 121 cases of posterior malleolus fractures [9]. This might be due to the fact that only two observers classified the CT scans in the former study, since Raeder et al. (with three observers) also found substantial interobserver reliability [9, 14].

The Mason classification for posterior malleolus fractures demonstrated only moderate interobserver reliability, whereas the Bartoníček classification had the best result. The intraobserver reliabilities for the different classification systems of the posterior malleolus also showed a substantial agreement with slightly higher kappa values. Mason et al. and Raeder et al. did not perform intraobserver analysis in their studies and therefore comparable results are missing [9, 14]. Our intraobserver reliabilities further endorse the findings of this study as valid and reproducible by providing evidence, that the assessment was not biased due to different investigators.

Bartoníček et al. proposed their CT-based classification system that takes into account the stability of the tibiotalar joint and the integrity of the fibular notch [8]. The Bartoníček classification system is currently endorsed in the literature and has led to classification-based treatment recommendations [7, 12, 21–23]. This is due to the fact that studies found a correlation between the Bartoníček type of posterior malleolus fracture and clinical outcome [22, 23]. In recent years, the trend goes to direct fixation of the posterior malleolus fragment, since it can provide a superior quality of reduction and better outcomes [11, 24, 25]. In this study, a larger posterior malleolus fragment led significantly more often to a fixation of the posterior malleolus, but no difference was found with respect to the influence of the size on the direct or indirect fixation method. This finding can be attributed to the observation period, spanning the time of 9 years and change of operative management due to new evidence of the benefit of direct fixation of the posterior malleolus in the last years [3]. Furthermore, the simplified view only considering the size of the fragment is insufficient, since fracture configuration with involvement of the fibular notch or medial malleolus are important influencing factors [11, 12]. In contrast to other studies, direct fixation of the posterior malleolus fragment did not supersede the necessity of a trans-syndesmotic screw. Miller et al. and Baumbach et al. demonstrated that direct fixation of the posterior malleolus can reduce the rate of syndesmotic instability [24, 26]. But, in a recent study of Raeder et al., all patients suffering from a AO 44-C fracture with involvement of the posterior malleolus were treated with a syndesmotic fixation [14]. The assessment of syndesmotic stability is sometimes challenging but crucial, since insufficient fixation can lead to adverse outcomes [27]. In the last 3 years of the observation period, direct fixation of the posterior malleolus was significantly more likely. In our cohort, this finding could not be explained by fracture size or the involvement of the fibular notch. Therefore, this can be interpreted as a result of increasing evidence of the benefits of direct fixation, that are supporting the trend regarding the treatment of fractures of the posterior malleolus in the last years [7, 11, 12].

Trimalleolar ankle fractures are a complex entity that can be described with the help of different classification systems. Today, there is no consensus which classification systems should be used routinely to guide treatment. New CT-based classifications led to a more differentiated view of the medial and posterior malleoli. At the moment, the focus is on the posterior malleolus, and treatment algorithms were derived from the Bartoníček classification. The results of this study support the use of the Bartoníček classification as a reliable tool, but further research has to investigate the possible influence of the type of medial and lateral malleoli fractures on outcomes. To especially be able to assess the outcomes after ankle fractures, it is essential to compare equivalent patient groups. The challenge is, on one hand, to take into account the new developments in research and, on the other hand, to agree on a standardized and rational methodology to code data for research and the clinic.

Limitations of the study are its retrospective design with its associated bias. Long-term clinical and radiological results were not part of our study; therefore, no assumption can be made about the prognostic value of the respective classification systems for the functional outcome. Due to the fact that only trimalleolar ankle fractures were included, certain subgroups of malleolar fractures can be underrepresented. Despite the limitations, the study includes the largest number of patients evaluating the reliability of classification systems in ankle fractures and analysed, for the first time, the reliability of the Bartoníček and Mason classifications of posterior malleolus fractures.

## Conclusion

In trimalleolar ankle fractures, the AO/OTA classification is a reliable system to characterize the type of fracture, but it fails to provide solid information about the medial and posterior malleoli. Nowadays, treatment recommendations for trimalleolar ankle fractures focus on the configuration of the posterior malleolus; therefore, the results of this study advocate the use of the Bartoníček classification as a reliable tool to guide treatment.

## Data Availability

The datasets generated and analysed during the current study are available from the corresponding author on request.

## References

[1] Heckman JD, McKee M, McQueen MM, Ricci W, Tornetta P (2014). Rockwood and green's fractures in adults.

[2] Elsoe R, Ostgaard SE, Larsen P (2018). Population-based epidemiology of 9767 ankle fractures. Foot Ankle Surg.

[3] Pflüger P, Braun K-F, Mair O, Kirchhoff C, Biberthaler P, Crönlein M (2021). Current management of trimalleolar ankle fractures. EFORT Open Rev.

[4] Meinberg E, Agel J, Roberts C, Karam MD, Kellam J (2018). Fracture and dislocation classification compendium—2018. J Orthop Trauma.

[5] Herscovici D, Scaduto J, Infante A (2007). Conservative treatment of isolated fractures of the medial malleolus. J Bone Jt Surg Br Vol.

[6] Carter T, Duckworth A, White T (2019). Medial malleolar fractures: current treatment concepts. Bone Jt J.

[7] Rammelt S, Bartonícek J (2020). Posterior malleolar fractures: a critical analysis review. JBJS Rev.

[8] Bartoníček J, Rammelt S, Kostlivý K, Vaněček V, Klika D, Trešl I (2015). Anatomy and classification of the posterior tibial fragment in ankle fractures. Arch Orthop Trauma Surg.

[9] Mason LW, Marlow WJ, Widnall J, Molloy AP (2017). Pathoanatomy and associated injuries of posterior malleolus fracture of the ankle. Foot Ankle Int.

[10] Haraguchi N, Haruyama H, Toga H, Kato F (2006). Pathoanatomy of posterior malleolar fractures of the ankle. JBJS.

[11] Verhage SM, Hoogendoorn JM, Krijnen P, Schipper IB (2018). When and how to operate the posterior malleolus fragment in trimalleolar fractures: a systematic literature review. Arch Orthop Trauma Surg.

[12] Vacas-Sánchez E, Olaya-González C, Abarquero-Diezhandino A, Sánchez-Morata E, Vilá-Rico J (2020). How to address the posterior malleolus in ankle fractures? A decision-making model based on the computerised tomography findings. Int Orthop.

[13] Aitken SA, Johnston I, Jennings AC, Chua IT, Buckley RE (2017). An evaluation of the Herscovici classification for fractures of the medial malleolus. Foot Ankle Surg.

[14] Ræder BW, Andersen MR, Madsen JE, Jacobsen SB, Frihagen F, Figved W (2021). Prognostic value of the Haraguchi classification in posterior malleolar fractures in A0 44-C type ankle fractures. Injury.

[15] Landis JR, Koch GG (1977). The measurement of observer agreement for categorical data. Biometrics.

[16] Malek I, Machani B, Mevcha A, Hyder N (2006). Inter-observer reliability and intra-observer reproducibility of the Weber classification of ankle fractures. J Bone Jt Surg Br Vol.

[17] Kennedy J, Johnson S, Collins A (1998). An evaluation of the Weber classification of ankle fractures. Injury.

[18] Hintermann B, Regazzoni P, Lampert C, Stutz G, Gächter A (2000). Arthroscopic findings in acute fractures of the ankle. J Bone Jt Surg Br Vol.

[19] Lübbeke A, Salvo D, Stern R, Hoffmeyer P, Holzer N, Assal M (2012). Risk factors for post-traumatic osteoarthritis of the ankle: an eighteen year follow-up study. Int Orthop.

[20] Liu Y, Lu H, Xu H (2021). Characteristics and classification of medial malleolar fractures: a study based on CT fracture mapping. Bone Jt J.

[21] Bartoníček J, Rammelt S, Tuček M (2017). Posterior malleolar fractures: changing concepts and recent developments. Foot Ankle Clin.

[22] Maluta T, Samaila E, Amarossi A (2021). Can treatment of posterior malleolus fractures with tibio-fibular instability be usefully addressed by Bartonicek classification?. Foot Ankle Surg.

[23] Neumann AP, Rammelt S (2021). Ankle fractures involving the posterior malleolus: patient characteristics and 7-year results in 100 cases. Arch Orthop Trauma Surg.

[24] Baumbach S, Herterich V, Damblemont A, Hieber F, Böcker W, Polzer H (2019). Open reduction and internal fixation of the posterior malleolus fragment frequently restores syndesmotic stability. Injury.

[25] Ribeiro HM, Silva J, Teixeira R, Fernandes P, Sobral L, Rosa I (2020). Clinical outcomes and trans-syndesmotic screw frequency after posterior malleolar fracture osteosynthesis. Injury.

[26] Miller MA, McDonald TC, Graves ML (2018). Stability of the syndesmosis after posterior malleolar fracture fixation. Foot Ankle Int.

[27] Yang T-C, Tzeng Y-H, Wang C-S, Lin C-C, Chang M-C, Chiang C-C (2020). Untreated small posterior fragment of ankle fracture with early removal of syndesmotic screw is associated with recurrent syndesmotic instability. Injury.

